# A Method to Assess the Relevance of Nanomaterial Dissolution during Reactivity Testing

**DOI:** 10.3390/ma13102235

**Published:** 2020-05-13

**Authors:** Willie J. G. M. Peijnenburg, Emmanuel Ruggiero, Matthew Boyles, Fiona Murphy, Vicki Stone, Derek A. Elam, Kai Werle, Wendel Wohlleben

**Affiliations:** 1 National Institute of Public Health and the Environment (RIVM), Center for Safety of Substances and Products, 3721 MA Bilthoven, The Netherlands; willie.peijnenburg@rivm.nl; 2 Institute of Environmental Sciences (CML), Leiden University, P.O. Box 9518, 2300 RA Leiden, The Netherlands; 3 Department of Material Physics & Analytics & Formulation, BASF SE, Carl-Bosch-Strasse 38, 67056 Ludwigshafen, Germany; emmanuel.ruggiero@basf.com (E.R.); derek-alexander.elam@basf.com (D.A.E.); Kai.Werle@basf.com (K.W.); 4 Institute of Occupational Medicine (IOM), Research Avenue North, Heriot-Watt University, Midlothian, Edinburgh EH14 4AP, UK; Matthew.boyles@iom-world.org; 5 Nano Safety Research Group, Heriot-Watt University, Edinburgh EH14 4AS, UK; f.murphy@hw.ac.uk (F.M.); v.stone@hw.ac.uk (V.S.)

**Keywords:** nanoform, grouping, nanoform dissolution, reactivity assay, reactivity class, dissolution product

## Abstract

The reactivity of particle surfaces can be used as a criterion to group nanoforms (NFs) based on similar potential hazard. Since NFs may partially or completely dissolve over the duration of the assays, with the ions themselves inducing a response, reactivity assays commonly measure the additive reactivity of the particles and ions combined. Here, we determine the concentration of ions released over the course of particle testing, and determine the relative contributions of the released ions to the total reactivity measured. We differentiate three classes of reactivity, defined as being (A) dominated by particles, (B) additive of particles and ions, or (C) dominated by ions. We provide examples for each class by analyzing the NF reactivity of Fe_2_O_3_, ZnO, CuO, Ag using the ferric reduction ability of serum (FRAS) assay. Furthermore, another two reactivity tests were performed: Dichlorodihydrofluorescin diacetate (DCFH_2_-DA) assay and electron paramagnetic resonance (EPR) spectroscopy. We compare assays and demonstrate that the dose-response may be almost entirely assigned to ions in one assay (CuO in DCFH_2_-DA), but to particles in others (CuO in EPR and FRAS). When considering this data, we conclude that one cannot specify the contribution of ions to NF toxicity for a certain NF, but only for a certain NF in a specific assay, medium and dose. The extent of dissolution depends on the buffer used, particle concentration applied, and duration of exposure. This culminates in the DCFH_2_-DA, EPR, FRAS assays being performed under different ion-to-particle ratios, and differing in their sensitivity towards reactions induced by either ions or particles. If applied for grouping, read-across, or other concepts based on the similarity of partially soluble NFs, results on reactivity should only be compared if measured by the same assay, incubation time, and dose range.

## 1. Introduction

There is already a high tonnage of nanoforms (NFs) commercially available, including fillers and pigments [1], with more under development, with applications ranging from electronics, coatings, clothing, food packaging, construction materials, and cosmetics [2]. Despite the need to register NFs to REACH from 2020 [3], the debate on which parameters are most appropriate for assessing the NF hazard to biological systems is still ongoing. Due to the large variety of current and future NFs, a grouping approach of NFs would be beneficial to avoid case-by-case risk assessment and to predict their adverse effects without animal testing. Most scientific frameworks of grouping consider surface-induced reactivity as a key property to rationalize and compare the toxicity of NFs [4,5,6,7,8,9,10]. Recently, the European Chemicals Agency (ECHA) published the registration and the grouping of NFs, which recommended “biological (re)activity” as one means of substantiating biological similarities [11]. Such guidance states that the reactivity of NFs can be modulated by each of the four properties that describe a NF: Size, shape, surface treatment, and specific surface area [12]. 

NFs can generate reactive oxygen species (ROS) that are sources of oxidative stress and cytotoxicity. ROS are powerful oxidants that indiscriminately damage cellular targets and can be generated through interaction with a particle surface by a variety of mechanisms: Fenton reaction, redox cycling, radical generation [13]. The ability of NFs to generate ROS can be defined as oxidative potential. Several analytical procedures, so called reactivity assays, are routinely used to determine NF oxidative potential and quantify the related ROS. Such reactivity assays generally involve the immersion of NFs in an aqueous medium in the presence of a probe which interacts with the ROS produced, thereby revealing the oxidative potential of the NFs by spectroscopic, spectrophotometric, or chromatographic methods [14]. 

However, some NFs may partially or completely dissolve to the ionic forms during the incubation time of the assays. In 2018, Gray et al. proposed a framework for grouping two-dimensional NFs based on their dissolution behavior in media. NF dissolution kinetics and their dissolved products are used to place NFs in 4 different classes with higher or lower priority for additional nanotoxi-cology testing [15].

It remains an open question therefore as to whether the measured reactivity derives from the reactive processes on the particle surface or whether it originates from interactions with dissolved ions. Especially for ZnO and CuO particles, the debate has been recurring over whether or not the observed reactivity is due to the ions or due to the remaining particles [6,16]. Grouping of NFs by their “surface reactivity” or “oxidative potential” is not robust without knowing the main contributor (the particle or the ions) for ROS generation.

In the simplest approach to assess reactivity of a NF, the characteristics that govern the reactivity of a suspension of soluble NFs, include chemical composition, diameter, shape, dissolution rate in a specific medium, reactivity of particle-surface, and reactivity of ions (or other dissolution products). Additionally, as different measures of reactivity use different protocols, dispersion media, and timing (from minutes to hours), all of the substance-related and method-related parameters that can influence the extent of particle dissolution during the assay need to be considered.

Here, we evaluate the oxidative potential of four particles (CuO, ZnO, Fe_2_O_3_, Ag) and their free ions utilizing the highly sensitive ferric reduction ability of serum (FRAS) assay. These particles are commonly used in a variety of applications ranging from electronic industries and medical devices to clothing and soil remediation [17]. Due to the expanding number of applications for each of these materials, there is an increased potential for release into the environment, inducing concern related to their possible adverse effects. The FRAS assay employs human serum and quantifies the total antioxidant depletion induced by NF as a measure of their oxidative potential. Considering additive contributions of particles and ions, we describe a model that differentiates three classes of reactivity: (A) Dominated by particles, (B) Additive of particles and ions, or (C) Dominated by ions, and demonstrate the implementation of this model using a pragmatic experimental workflow. Selecting one NF (CuO), we also compare the relative contributions of particles and ions established by the FRAS assay with two further well-established acellular assays: The acellular dichlorodihydrofluorescein diacetate (DCFH_2_-DA) assay and electron paramagnetic resonance (EPR) spectroscopy with each test differing greatly in principle and experimental methodology. The DCFH_2_-DA assay determines the oxidation of non-fluorescent molecules into a fluorescent form in presence of ROS, whereas EPR is a standardized technique to identify and quantify free radicals via spin trap [14]. The aim of the research performed was to compare the impact of particles and ions on the performance of three assays for assessing the reactivity of suspensions of NFs. Interestingly, the results reported reveal differences in the relative contribution of particles and ions to the reactivity measurements, which may be a result of methodological differences in incubation time, dispersion media, etc. Therefore, we recommend that care should be taken when assigning the relative contribution of particles and ions to reactivity measurement and suggest that when conducting reactivity assays for grouping or read-across purposes, results on reactivity should only be compared if measured by the same assay, incubation time, and dose range.

## 2. Materials and Methods

### 2.1. Materials

Two NFs (CuO and Fe_2_O_3_ nano_A) were obtained from industries and two (Ag NM300k and ZnO NM111) were received from the European Joint Research Center (JRC, Ispra, Italy). The coding used equals the JRC code. CuO NF was purchased by PlasmaChem (Berlin, Germany). Fe_2_O_3_ nano_A pigment was supplied by BASF Colors and Effects (Ludwigshafen, Germany). Ag NM300k and ZnO NM111 were obtained from the JRC nanomaterial repository [18]. Such repository includes representative industrial NFs studied in large research projects and in the Organization for Economic Co-operation and Development testing program. Metal salts were provided by EMSURE (ZnCl_2_ and CuSO_4_·5H_2_O, Darmstadt, Germany), Honeywell Fluka (AgNO_3_, Seelze, Germany) and Merk (FeSO_4_·7H_2_O, Darmstadt, Germany).

The physicochemical properties of Ag NM300k [19] and the other NFs [9] have been previously reported and are included in the supplementary information (Table S1). The reagents employed during each reactivity assay were reported in the supplementary information.

### 2.2. FRAS

In FRAS, antioxidants in human blood serum (HBS) act as “reporters” for quantifying the oxidative damage induced by NFs [20]. The depletion of the total antioxidants in HBS due to interaction with ROS produced by NFs is quantified using the Fe-2,4,6-tripyridyl-s-triazine (TPTZ) complex. The method is based on the reduction of Fe(III)-TPTZ complex to a Fe(II)-TPTZ by residual antioxidant components present in HBS after NF exposure. A detailed protocol of the assay has been reported elsewhere [21]. In short, the test is divided in three steps: (I) The preincubation of the NFs (0.02–38 g/L) with HBS for 3 h at 37 °C, (II) the separation of the NFs from HBS via ultracentrifugation step (AUC-Beckman XL centrifuge (Brea, CA, USA) at 140,000 G for 150 min) and (III) the transfer of 100 μL of NF-free HBS supernatant to the FRAS reagent solution that contains the Fe(III)-TPTZ complex. Thus, the UV-vis spectrum of the iron complex solution is recovered to determine the total antioxidant depletion as a measure of the oxidative potential of NFs. Trolox, a water-soluble analog of vitamin E, was used as an antioxidant to calibrate the FRAS results. FRAS protocol was performed at various Trolox concentration (from 0.001 to 0.1 g/L) for obtaining FRAS absorption signals that can be linearly fitted. Thus, the oxidative damage induced by NFs was then calculated in Trolox equivalent units (TEUs).

Additionally, fresh NF samples were prepared to evaluate the ion contribution. After an ultracentrifugation step, the ion concentration in NF-free HBS supernatants was determined by ICP-MS (Perkin Elmer, Nexion 2000b, Waltham, MA, USA). Using water soluble metal salts (ZnCl_2_, CuSO_4_·5H_2_O, AgNO_3_, FeSO_4_·7H_2_O), ions solutions with equivalent concentrations were prepared and the associated oxidative damage in HBS was measured by FRAS method. For each NF and ions dose, triplicate measurements were performed.

### 2.3. EPR

EPR spectroscopy was employed to detect hydroxyl radicals (^•^OH) using a DMPO spin trap. Applying the standard ISO TS18827:2017, measurements were performed on a Bruker EMXnano (CW, X-band, Elk Grove Village, IL, USA) incubating CuO NFs (1–4000 mg/L) for 30 min in deionized water containing 50 mM DMPO, 0.001 mM FeSO_4_, and 0.01 mM H_2_O_2_. Table S2 in the supplementary reports the list of key parameters used during the analysis. 

Once CuO reactivity was determined, another solution of CuO was prepared, incubated, and centrifuged (Heraeus Sepatech centrifuge, Dreieich, Germany) at 5600 G for 10 min. ICP-MS was used to determine the Cu ion concentration in the supernatant. A solution of CuSO_4_ with equivalent Cu concentration was prepared and measured with EPR spectroscopy to determine ion reactivity.

### 2.4. DCFH_2_-DA

To determine the contribution of Cu ions to the oxidation of the 2′-7′-dichlorodihydrofluorescin diacetate (DCFH_2_-DA) probe, first the DCFH_2_-DA assay was performed with use of particles only. DCFH_2_-DA was prepared by an initial step of chemical hydrolysis of DCFH_2_ to DCFH-DA in 0.01 M NaOH. Once neutralized, DCFH_2_-DA was diluted to 10 µM in phosphate-buffered saline (PBS). CuO NFs were suspended in phenol red-free minimum essential medium (MEM) with 2% FCS at a concentration of 1 g/L. This stock was sonicated for 15 min in an ultra-sonicating water bath prior to serial dilution. Each treatment was added, in triplicate, at a volume of 25 µL to a 96-well plate, followed by the addition of 225 µL of the 10 µM DCFH_2_-DA reagent, giving a final CuO concentration of 1.6, 3.1, 6.3, 12.5, 25, 50, 100 mg/L. Fluorescence intensity was measured at 485/530 ex/em after incubation at 37 °C for 0, 30, 60, and 90 min. The data presented is of measurements at either 30 or 90 min minus measurement at 0 min.

Once these data were collected, two samples were prepared to represent the ionic fraction released from CuO NFs during the course of the DCFH_2_-DA assay. These samples represent the onset of reactivity at EC20 (low dose) and the maximum CuO NF (high dose) concentration used. The onset of reactivity was determined to be 0.013 g/L (final concentration), and maximum concentration was 0.1 g/L (final concentration). These CuO NF concentrations were suspended and handled as per the protocol described above, using two incubation times of 90 and 30 min. Each sample was subsequently centrifuged at 75,000 G for 30 min and the supernatant was removed for Cu quantification by ICP-OES. At 90 min, the equivalent to 2.5 mg/L was released from the low dose, and 7.0 mg/L from the high dose; at 30 min, 1.7 and 5.9 mg/L were released from the low and high dose, respectively. Using corresponding copper concentrations from copper sulphate (CuSO_4_·5H_2_O), the DCFH_2_-DA assay was performed as before, and data generated from Cu ions were compared to that of CuO NPs.

### 2.5. Workflow

**Figure 1:**
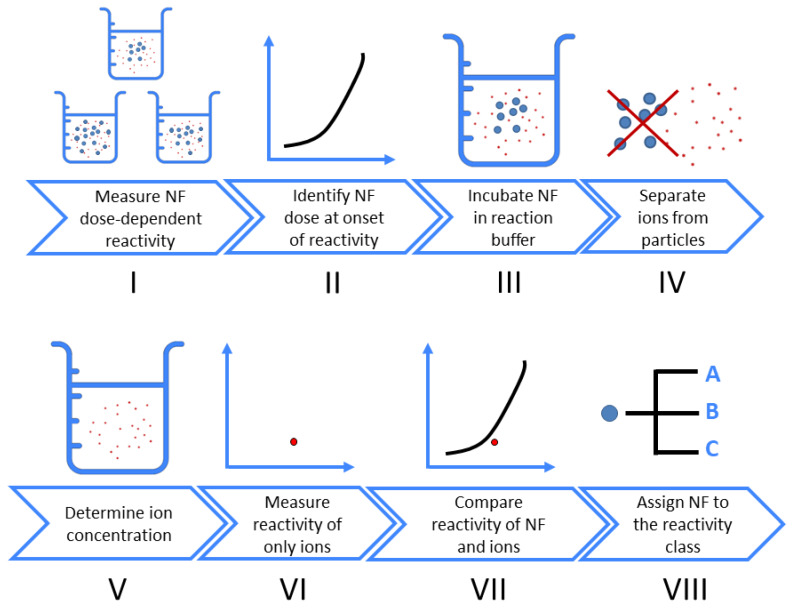
Stepwise workflow to assess whether ions contribute to dose-response curves of nanoform (NF) oxidative potential.

In this work, we design an experimental stepwise workflow (Figure 1) to determine the possible contribution of ion to the overall NF oxidative potential. First, the dose-reactivity in response to the NF is measured. Within this dose range, the concentration of NF which induces the onset of a response (e.g., at ~20% of the maximum damage) is established, and is used to determine the concentration of released ions at the end of the incubation time, using sensitive analytical methods (e.g., ICPMS, ICP-OES). A soluble salt is then used in place of the NF, at an identical metal ion concentration. It should be noted that this approach delivers a high bolus concentration of ions at the start of the reactivity assay, and does not exactly replicate the kinetic release of ions throughout the assay. The complete assay is performed to determine the reactivity of these ions. With a comparison of the ion response to that of the original NF response, one can classify this NF and attribute its oxidative potential to either the particle itself, only the released ions, or a combination of the two.

## 3. Results and Discussion

### 3.1. Concept of Reactivity Classes and Workflow

Considering the dissolution behavior of NFs in an aqueous medium, it is possible to assign NFs to classes based on the kinetics of dissolution, the reactivity of the NF in a specific reactivity assay, and the dissolution product(s), which are in most cases ions. For the specific case of closed batch systems, the thermodynamically driven force for dissolution will gradually decrease, resulting in a specific steady-state concentration of ions in suspension that will be dependent on the initial particle concentration. Hence, the ratio of particles-to-dissolution products at steady state will depend on the initial particle concentration.

The implications of these considerations are that the reactivity of a NF suspension, as measured in a specific reactivity assay, will depend on the initial particle concentration, the reactivity of the particles, the reactivity of the ions formed, and the rate of dissolution of the NF as affected by particle size and particle composition. Through consideration of dissolution kinetics and of the additive reactivity of particles and ions, we distinguish three classes of (partially or fully) soluble spherical NFs:

(A) Soluble NFs, for which suspension reactivity is determined by particle reactivity, either because the ions released are less reactive than the NF or because ion release kinetics are slow or because the solubility limit is below the ion effect threshold.
  (a) Most literature shows that a reduced toxicity of ions as compared to particle toxicity is the exception rather than the rule [22].
  (b) Examples include spherical SiO_2_ at pH above 6.
(B) Soluble NFs, for which both particles and ions contribute significantly to reactivity, depending on medium and exposure duration. Read-across of NFs is possible only after detailed analysis of rates of dissolution as well as information on the reactivity of NFs and ions.
  (a) Ag-particles in a system with apparent equilibration of ion release.
(C) Soluble NFs, for which suspension reactivity can be quantified by read-across using ion reactivity. This can be the consequence of two options: (I) The case in which reactivity of ions and particles is similar; (II) the case in which the concentration of released ions strongly exceeds the concentration of particles, whilst this does not discount by the possibility of the particles being far more reactive than the ions, the rate of dissolution is such that the ions are considered the primary source of reactivity.
  (a) Examples with regard to toxicity include Pb-based perovskites (very high rates of dissolution) and ZnO-nanoparticles of different size and shape (time-dependent kinetics, but in general toxicity of Zn-ions and Zn NFs similar).

Following the stepwise workflow (Figure 1), one can determine the contribution of inadvertently generated ions to the oxidative potential measured from an NF in suspension. 

If the particles dominate the reactivity against the ions, then the NF belongs to class A. Surface reactivity in this class is advised as a key property to assess the biological similarity of different NFs of the same substance.

When both particle and ions contribute significantly to the oxidative potential of the NF, the sample belongs to class B; both dissolution in the biological compartment and surface reactivity need to be considered in comparing NFs.

On the contrary, if the ions from the salt generate an assay response which, suggests that the ions predominantly determine the reactivity of the NF, the NF belongs to class C. In this case, the dissolution is the most important property to substantiate similarity between different NFs, whereas the reactivity assay is less relevant to compare NFs. 

Additionally, one may assess the ion concentration at several NF doses. If saturating, the solubility limit is reached for the specific medium and incubation time, and this may help to understand which part of the dose-response curve marks the onset of particle surface reactivity in addition to ion reactivity.

### 3.2. Implementation of Workflow on Different NFs in One Assay

We tested our concept and workflow in two scenarios. Firstly, the workflow was implemented with one assay (FRAS) to identify the class of four NFs (CuO, ZnO NM110, Fe_2_O_3_ nano_A, Ag NM300k). Secondly, we challenged the consistency of our classification by comparing three assays (FRAS, EPR, DCFH_2_-DA) with one NF (CuO).

**Figure 2:**
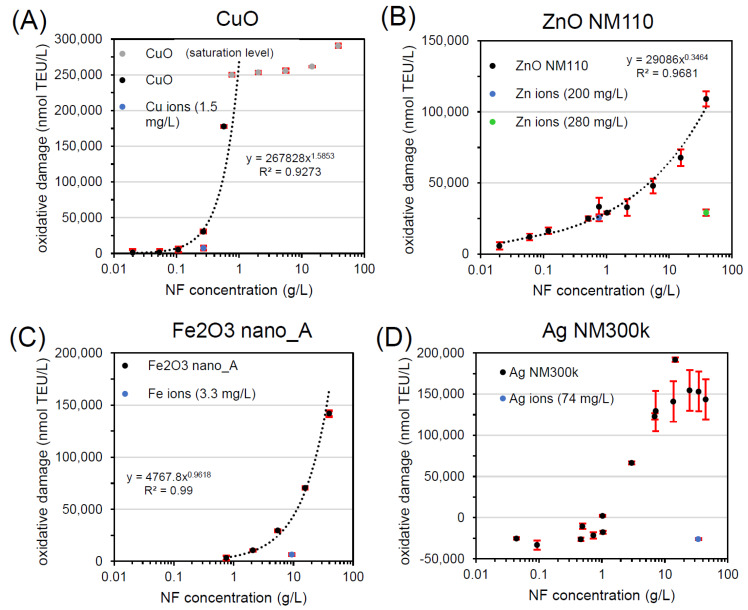
Workflow implementation with FRAS testing (**A**) CuO, (**B**) ZnO NM110, (**C**) Fe_2_O_3_ nano_A, and (**D**) Ag NM300k. All curves represent dose-response data of particles (black, grey) and of ions (blue, green). TEU, Trolox equivalent unit (mM).

Figure 2 and Table 1 show the results of workflow implementation with FRAS for all NFs. For each sample, a dose-response was carried out and one concentration close to ~20% of maximum NF oxidative potential was selected for further evaluation. CuO was the first NF tested (Figure 2A). After incubation of the CuO NF in the FRAS assay buffer/media for the duration of the assay at a concentration of 0.27 g/L (~20% of the measured CuO oxidative potential), the actual Cu ion concentration was determined: 0.017 mg/L. Only a fraction of 0.006% of the dosed particle mass was found in ionic form. Due to FRAS set-up limitations, the lowest concentration of Cu ion measurable was 1.5 mg/L, which is 2 orders of magnitude higher than the real ion concentration. Despite the overestimation, the ion oxidative potential was four times lower (7510 nmol TEU/L) than the response induced by the total CuO NF (30497 nmol TEU/L). The reactivity of CuO at 0.27 g/L was predominately assigned to the particle with a steep dose-response curve. Such curve displayed an exponential trend in the first part of the curve as demonstrated by the fitting equation in the graph. Accordingly, CuO in FRAS belongs to class A.

The ion contribution on ZnO NM110 was tested at two concentrations (Figure 2B), relevant to the following particle concentrations: 0.77 g/L (low dose close to onset of reactivity) and 38.8 g/L (high dose). At low NF concentration, the workflow revealed that ZnO NM110 reactivity at low dose originated completely from the ions, whose response superimposed the NF dose-response. At a high NF concentration of 38.8 g/L, we found contribution of both particles and ions. Since the test reached the solubility limit, the concentration of Zn ions did not change in the two conditions: 200 mg/L (low dose: 0.77 g/L ZnO) and 280 mg/L (high dose: 38.8 g/L ZnO). Depending of NF dose tested, ZnO NM110 in FRAS can be assigned to class C (low dose) or class B (high dose). Compared to expected physiological concentration, class C is relevant for the in vivo reactivity extrapolation.

For Fe_2_O_3_ nano_A, the FRAS assay presented significant reactivity with a steep dose-response with exponential trend (Figure 2C). Fe ion release was negligible with only 0.04% of dissolution and did not significantly affect the ferric reduction ability of serum. Thus, Fe_2_O_3_ in FRAS belongs to class A because the reactivity is fully attributed to particles.

Finally, the workflow was also implemented for Ag NM300k. The dose-response curve displayed an artefact of apparent negative Ag NM300k reactivity up to 1 g/L (Figure 2D). As reported in the literature [20,23], using high NF doses (1–10 g/L) is advisable to avoid false negatives. Our results can be raised by the reactive dissolution pathway of Ag NFs (Ag^0^ → Ag^+^ + e^−^). However, only 0.2% of total Ag NM300k amount was dissolved. Considering that Ag ions displayed negligible contribution to reactivity, Ag NM300k in FRAS was class A. 

These four examples demonstrate that the workflow makes the classes of the concept applicable to experimental results in a pragmatic manner.

### 3.3. Implementation of Workflow on One NF in Different Assays

In the second scenario, we focused on one NF (CuO). We compared the outcome of FRAS with the other two assays (EPR, DCFH_2_-DA) to test the consistency of CuO classification.

**Figure 3:**
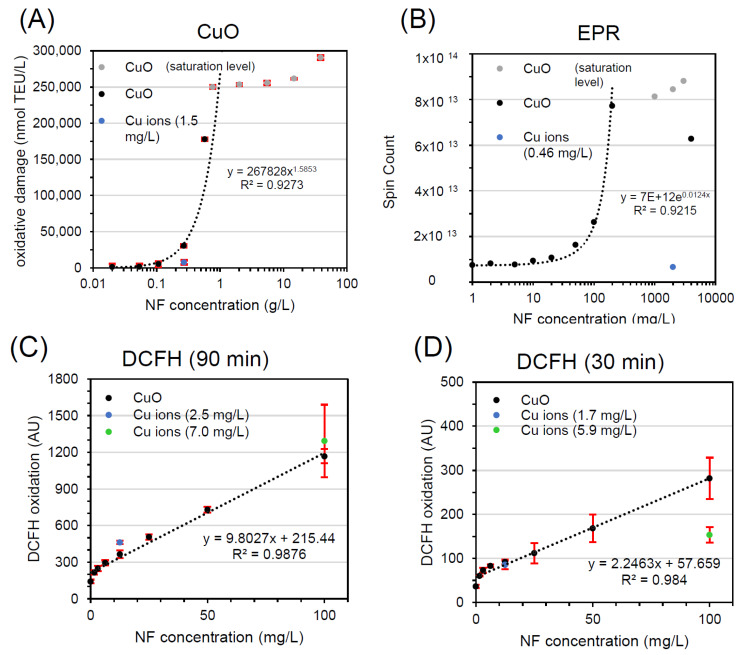
Workflow implementation with FRAS (**A**), EPR (**B**), DCFH_2_-DA (**C**,**D**) testing CuO NF and ion reactivity. All curves represent dose-response data of particles (black, grey) and of ions concentration (blue, green). TEU, Trolox equivalent unit (mM); AU, arbitrary unit.

The dose-response curve of EPR was steep, and reached saturation at 0.2 g/L (Figure 3B), similar to saturation in FRAS at 0.5 g/L (Figure 3A). The ion release, evaluated at high CuO dose (2 g/L), was determined to be low (0.46 mg/L), showing an ion-to-particle ratio comparable to FRAS assay. In such conditions CuO NFs concentration is 4 orders of magnitude higher than Cu ions alone. Consequently, Cu ions only weakly promoted the EPR:DMPO-OH signals and CuO was classified as A. 

Lastly, the contribution of Cu ions in DCFH_2_-DA oxidation, in comparison to CuO NPs, was tested (Figure 3C,D) at two NF concentrations: 12.9 mg/L (low dose = EC20) and 100 mg/L (high dose = maximum particle concentration used), and two different time points: 90 min (Figure 3C) and 30 min (Figure 3D). Unlike FRAS and EPR, Cu ions substantially contributed to NF reactivity in the DCFH_2_-DA assay at both concentrations. At the latter time point, the oxidation of DCFH appears to be driven by released Cu ions; at the earlier time point, this is also true for the low dose, however, at the high dose, it appeared both particles and ions contribute. A key difference is the dose range: the DCFH_2_-DA abiotic assay, often conducted in parallel to the use of DCFH_2_-DA in cell exposures, is usually performed at the low concentrations that are admissible to cell cultures: mg/L instead of the g/L used in the strictly abiotic EPR and FRAS methods. Hence, according to solubility limit, the share of particles that dissolve is higher in DCFH_2_-DA. In DCFH_2_-DA analysis of CuO, the ion-to-particle ratio (25% and 9% after 90 min, and 17% and 7% at 30 min) is 3 orders of magnitudes higher than in FRAS and EPR tests. But that is not the only difference: The CuO dose-response curve in DCFH_2_-DA has negative curvature at lower concentrations and display a linear trend as concentrations increase up to 0.1 g/L, whereas it has positive and exponential curvature (onset of particle reactivity) in EPR and FRAS at higher dose. CuO in DCFH_2_-DA was assigned to class C in most cases, because the Cu ion release is the main contributor to CuO reactivity, and to class B at high particle concentration (relative to assay restrictions) at early time points.

Nevertheless, it is important to highlight that the determination of the ion concentration at the end of incubation results in an extension of the contact time between the particles and the fluid and this, in combination with using a bolus dose of metal ions, may increase the release of ions with regards to correct ion concentration. Hence, the workflow may overestimate the contribution of ions during the NF incubations in FRAS and DCFH_2_-DA. The separation time was 150 min for FRAS and 30 min for DCFH_2_-DA respectively, but 10 min for EPR. However, there is evidence that Cu ions are generated during the test and have a strong contribution to the oxidation of DCFH_2_-DA. As it was thought plausible that the differences in effects observed between these three assays were due to the observations of low (FRAS, EPR) or high (DCFH_2_-DA) ion release in different medium, as opposed to differences in the way each assay interacts with particles and/or ions. As reported in the supplementary information (Figure S1), we have shown that FRAS would also have a high sensitivity to ions if present. 

In summary of measuring CuO in the three reactivity assays, the classification was not unequivocal. FRAS and EPR classified CuO as A, where the reactivity is determined by particles. Instead, DCFH_2_-DA demonstrated a strong ion contribution, mostly categorizing CuO as class C. 

This outcome can be elucidated taking in consideration three main factors: (I) Diverse assays analyzed different NF dose concentration (from mg/L to g/L), because the probability that the particle surfaces contribute to reactivity is higher if tested at a concentration above the solubility limit, (II) the DCFH_2_-DA buffer leads to higher CuO dissolution than the EPR and FRAS ones, and (III) assays may display different sensitivity to ions or particles. As results, DCFH_2_-DA assay seems to be especially dominated by Cu ions, whereas the FRAS assay is sensitive to Cu ions, but still dominated by particle-induced reactivity. In comparison to ZnO in FRAS, the classes B or C apply depending on the dose tested. The literature to ZnO and CuO has often tried to assign the nanomaterial to either class A, B, or C, but our results caution such assignment to material properties, and instead recommend an assignment for the combination of assay (incl. medium, dose range etc.) and material.

## 4. Conclusions

In this work, we present a practical workflow to determine the contribution to reactivity by particle surfaces and by ions that are inadvertently generated during incubation of NFs in an aquatic medium. Up till now, it has been overlooked that NFs as well as ions contribute to the outcome of reactivity assays. The workflow that we present is new as it for the first time allows the contribution of NFs and ions to reactivity to be quantified. Amongst others, the underlying concept of separation of the contributions of NFs and ions is broadly applicable to any reactivity assay as well as to, for instance, toxicity assessment of aquatic suspensions of NFs.

We find that the “relative” toxicity of ions and particles may be a misleading concept. Because the incubation phases of assays differ with regard to medium, concentration, and duration, the DCFH_2_-DA, EPR, FRAS assays are performed under different ion-to-particle ratios. Additionally, the assays differ in their sensitivity towards reactions induced by either ions or particles. Taken together, the dose-response to CuO nanoparticles may be assigned mostly to ions in one assay (DCFH_2_-DA), but almost entirely to particles in another (FRAS). 

If applied for grouping, read-across, or other concepts based on the similarity of NFs, results on reactivity should only be compared if measured by the same assay, incubation time, and dose range. For the specific case of ZnO in FRAS and for CuO in DCFH_2_-DA, the reactivity at doses up to 100 µg/mL is mostly assigned to the ions. For these materials, similarity of dissolution kinetics may be more important to compare different NFs of the same substance than testing their reactivity.

## Figures and Tables

**Table 1:** Summary of reactivity data for NFs and related ions in the three assays.

NF	Assay	NF Conc. (g/L)	Total Assay Reactivity	Ion Conc. (mg/L)	Ion Release (% of Total Cu Content)	Ion Contribution to Reactivity in Relation to Particle Response	Class
CuO	FRAS	0.27	30,497 ± 1910 nmol TEU/L	0.017	0.006	<<25% *	A
	EPR	2.0	8.45 × 10^13^ Spin count	0.46	0.023	7.8%	A
	DCFH_2_-DA (90 min)	12.5 × 10^−3^	364 ± 32 AU	2.5	24.9	127%	C
		100 × 10^−3^	1168 ± 58 AU	7.0	8.8	111%	C
	DCFH_2_-DA (30 min)	12.5 × 10^−3^	92 ± 5 AU	1.7	16.7	93%	C
		100 × 10^−3^	282 ± 47 AU	5.9	7.4	54%	B
ZnO NM110	FRAS	0.77	33,252 ± 6470 nmol TEU/L	200	26	92%	C
		38.8	109,075 ± 5297 nmol TEU/L	280	0.72	25%	B
Fe_2_O_3_ nano_A		9.4	41,141 ± 426 nmol TEU/L	3.3	0.035	16%	A
Ag NM300k		34.6	153,010 ± 24,221 nmol TEU/L	74	0.21	n.a.	A

* The oxidative potential of Cu ions (1.5 mg/L) measures 7510 nmol TEU/L.; TEU, Trolox equivalent unit (mM); AU, arbitrary unit.

## Supplementary Materials

The following are available online at https://www.mdpi.com/1996-1944/13/10/2235/s1. Figure S1: FRAS dose-dependent results for Cu ions, Table S1: Main physicochemical characteristics of NFs, Table S2: The list of key parameters employed during EPR measurements.

## References

[1] (2015). Ministère de l’Environnement, Éléments Issus des Déclarations des Substances à L’État Nanoparticulaire: Exercice 2015.

[2] Wohlleben W., Punckt C., Aghassi-Hagmann J., Siebers F., Menzel F., Esken D., Drexel C.-P., Zoz H., Benz H.U., Weier A., Mansfield E., Kaiser D.L., Fujita D., Voorde M.V.d. (2017). Nanoenabled Products: Categories, Manufacture, and Applications: Protocols and Industrial Innovations. Metrology and Standardization for Nanotechnology: Protocols and Industrial Innovations.

[3] The European Commission, The European Commission (2018). Commission Regulation (EU) 2018/1881 of 3 December 2018 amending Regulation (EC) No 1907/2006 of the European Parliament and of the Council on the Registration, Evaluation, Authorisation and Restriction of Chemicals (REACH) as regards Annexes I, III, VI, VII, VIII, IX, X, XI, and XII to address nanoforms of substances (Text with EEA relevance.). C/2018/7942.

[4] Oomen A.G., Steinhäuser K.G., Bleeker E.A.J., van Broekhuizen F., Sips A., Dekkers S., Wijnhoven S.W.P., Sayre P.G. (2018). Risk assessment frameworks for nanomaterials: Scope, link to regulations, applicability, and outline for future directions in view of needed increase in efficiency. NanoImpact.

[5] Oomen A.G., Bleeker E.A., Bos P.M., van Broekhuizen F., Gottardo S., Groenewold M., Hristozov D., Hund-Rinke K., Irfan M.-A., Marcomini A. (2015). Grouping and read-across approaches for risk assessment of nanomaterials. Int. J. Environ. Res. Public Health.

[6] Zhang H., Ji Z., Xia T., Meng H., Low-Kam C., Liu R., Pokhrel S., Lin S., Wang X., Liao Y.P. (2012). Use of Metal Oxide Nanoparticle Band Gap to Develop a Predictive Paradigm for Oxidative Stress and Acute Pulmonary Inflammation. ACS Nano.

[7] Arts J.H.E., Hadi M., Irfan M.-A., Keene A.M., Kreiling R., Lyon D., Maier M., Michel K., Petry T., Sauer U.G. (2015). A decision-making framework for the grouping and testing of nanomaterials (DF4nanoGrouping). Regul. Toxicol. Pharmacol..

[8] Hund-Rinke K., Schlich K., Kühnel D., Hellack B., Kaminski H., Nickel C. (2018). Grouping concept for metal and metal oxide nanomaterials with regard to their ecotoxicological effects on algae, daphnids and fish embryos. NanoImpact.

[9] Wohlleben W., Hellack B., Nickel C., Herrchen M., Hund-Rinke K., Kettler K., Riebeling C., Haase A., Funk B., Kühnel D. (2019). The nanoGRAVUR framework to group (nano)materials for their occupational, consumer, environmental risks based on a harmonized set of material properties, applied to 34 case studies. Nanoscale.

[10] McCarrick S., Wei Z., Moelijker N., Derr R., Persson K.-A., Hendriks G., Odnevall Wallinder I., Hedberg Y., Karlsson H.L. (2019). High variability in toxicity of welding fume nanoparticles from stainless steel in lung cells and reporter cell lines: The role of particle reactivity and solubility. Nanotoxicology.

[11] (2019). Appendix, R.6-1 for Nanoforms Applicable to the Guidance on QSARs and Grouping of Chemicals.

[12] (2019). Appendix for Nanoforms Applicable to the Guidance on Registration and Substance Identification.

[13] Bi X., Westerhoff P. (2019). Ferric reducing reactivity assay with theoretical kinetic modeling uncovers electron transfer schemes of metallic-nanoparticle-mediated redox in water solutions. Environ. Sci. Nano.

[14] Hellack B., Nickel C., Albrecht C., Kuhlbusch T.A.J., Boland S., Baeza-Squiban A., Wohlleben W., Schins R.P.F. (2017). Analytical methods to assess the oxidative potential of nanoparticles: A review. Environ. Sci. Nano.

[15] Gray E.P., Browning C.L., Wang M., Gion K.D., Chao E.Y., Koski K.J., Kane A.B., Hurt R.H. (2018). Biodissolution and cellular response to MoO 3 nanoribbons and a new framework for early hazard screening for 2D materials. Environ. Sci. Nano.

[16] Anders C.B., Eixenberger J.E., Franco N.A., Hermann R.J., Rainey K.D., Chess J.J., Punnoose A., Wingett D.G. (2018). ZnO nanoparticle preparation route influences surface reactivity, dissolution and cytotoxicity. Environ. Sci. Nano.

[17] Stark W., Stoessel P., Wohlleben W., Hafner A. (2015). Industrial applications of nanoparticles. Chem. Soc. Rev..

[18] JRC Nanomaterials Repository. https://ec.europa.eu/jrc/en/scientific-tool/jrc-nanomaterials-repository.

[19] Maier G., Romazanov J., Krug H., Hund-Rinke K., Locoro G., Wick P., Kuhlbusch T.A.J., Werner J., Mast J., Kördel W. (2011). NM-Series of Representative Manufactured Nanomaterials. NM-300 Silver Characterisation, Stability, Homogeneity—Study.

[20] Hsieh S.-F., Bello D., Schmidt D.F., Pal A.K., Stella A., Isaacs J.A., Rogers E.J. (2013). Mapping the Biological Oxidative Damage of Engineered Nanomaterials. Small.

[21] Gandon A., Werle K., Neubauer N., Wohlleben W. (2017). In Surface reactivity measurements as required for grouping and read-across: An advanced FRAS protocol. J. Phys. Conf. Ser..

[22] Hua J., Vijver M.G., Ahmad F., Richardson M.K., Peijnenburg W.J.G.M. (2014). Toxicity of different-sized copper nano- and submicron particles and their shed copper ions to zebrafish embryos. Environ. Toxicol. Chem..

[23] Pal A., Hsieh S.-F., Khatri M., Isaacs J., Demokritou P., Gaines P., Schmidt D., Rogers E., Bello D. (2014). Screening for oxidative damage by engineered nanomaterials: A comparative evaluation of FRAS and DCFH. J. Nanopart. Res..

