# Supplementary material for: A Method to Assess the Relevance of Nanomaterial Dissolution during Reactivity Testing
Source: Materials (Basel). 2020 May 13;13(10):2235. doi: 10.3390/ma13102235 (PMC7288060; doi:10.3390/ma13102235)
Supplement: Supplementary file 1 [file materials-13-02235-s001.pdf]

materials

MDPI

Supplementary Materials

A Method to Assess the Relevance of Nanomaterial Dissolution During Reactivity Testing

Willie J.G.M. Peijnenburg [1,2,†], Emmanuel Ruggiero [3,†], Matthew Boyles [4], Fiona Murphy [5], Vicki Stone [5], Derek A. Elam [3], Kai Werle [3] and Wendel Wohlleben [3,*]

1. National Institute of Public Health and the Environment (RIVM), Center for Safety of Substances and Products, 3721 MA Bilthoven, The Netherlands; willie.peijnenburg@rivm.nl
2. Institute of Environmental Sciences (CML), Leiden University, P.O. Box 9518, 2300 RA Leiden, The Netherlands
3. Department of Material Physics & Analytics & Formulation, BASF SE, Carl-Bosch-Strasse 38, 67056 Ludwigshafen, Germany; emmanuel.ruggiero@basf.com (E.R.); derek-alexander.elam@basf.com (D.A.E.); Kai.Werle@basf.com (K.W.)
4. Institute of Occupational Medicine (IOM), Research Avenue North, Heriot-Watt University, Midlothian EH14 4AP Edinburgh, UK; Matthew.boyles@iom-world.org
5. Nano Safety Research Group, Heriot-Watt University, EH14 4AS Edinburgh, UK; f.murphy@hw.ac.uk (F.M.); v.stone@hw.ac.uk (V.S.)

* Correspondence: wendel.wohlleben@basf.com

† Co-first authors

Received: 23 March 2020; Accepted: 7 May 2020; Published: date

1. Reactivity assay reagents

Below, we reported the list of materials used during each assay. In the FRAS assay, the materials employed were: sodium acetic trihydrate (Sigma Aldrich BioUltra, Darmstadt, Germany), glacial acetic acid (Alfa Aesar, Haverhill, MA, USA), 2,4,6-tri(2-pyridyl)-s-triazine (Sigma Aldrich, purity: ≥98%), HCl (Riedel-De Haen, Seelze, Germany, concentration: 1M), $FeCl_3 \cdot 6H_2O$ (Sigma Aldrich), human blood serum (Sigma Aldrich). In EPR spectroscopy, the materials used were: 5,5-Dimethyl-1-pyrroline-N-oxide (Enzo Life Sciences ALX-430-090, Oyster Bay, NY, USA), hydrogen peroxide (H_2O_2, 30%, Suprupus K49549298 824, Sigma Aldrich). In the DCFH test, we employed the following materials: 2′,7′-dichlorodihydrofluoresin diacetate (Sigma Aldrich), fluorescein diacetate (Sigma Aldrich) NaOH (Sigma Aldrich), methanol (analytical grade) (Sigma Aldrich), 0.1M (×10) PBS (Gibco DPBS 10×, Gaithersburg, MD, USA) phenol red free MEM (Gibco), and FCS (heat inactivated, Gibco).

Table S1. Main physicochemical characteristics of NFs.

NF	CAS	TEM Picture	Primary Particle Dimension (TEM)	Surface Area (BET)	Surface Chemistry (XPS)
CuO	1317-38-0	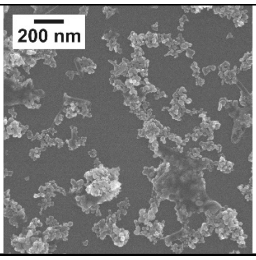	24 nm	34	7% C, 47% O, 46% metals

ZnO NM110	1314-32-2	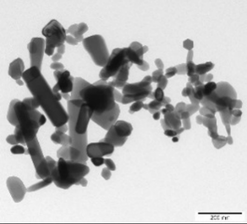	42 nm	12	30% C, 38% O, 38% metals, 3% non metals
Fe_2O_3 nano_A	1309-86-9	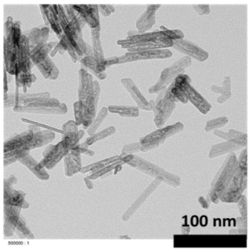	12 nm	107	16% C, 54% O, 28% metals, 1.8% non metals
Ag NM300k	7440-22-4	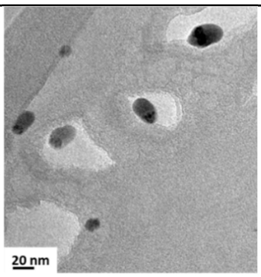	7.2 nm	n.a.	66% C, 29% O, 1.9% metals, 3.1% non metals

Table S2. The list of key parameters employed during EPR measurements.

Parameter	Bruker
Center field	3440 G
Range	100 G
Sweep time	100 s
Time constant	0.07–40.96 ms
#points	4100
Modulation amplitude	1 G
Modulation frequency	100 KHz
Microwave Attenuation	10 dB (10 mW power)
Receiver Gain	30–60 dB
#scans	16
Digital filter	Manual, 0 points
smooth	0–4 points

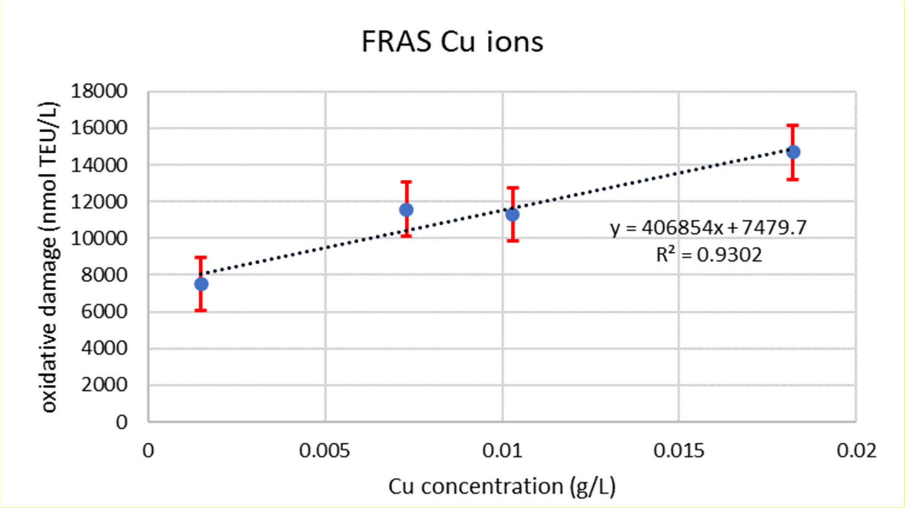

Figure S1. FRAS dose-dependent results for Cu ions.
